# Updating the probability of study success for combination therapies using related combination study data 

**DOI:** 10.1177/09622802231151218

**Published:** 2023-02-12

**Authors:** Emily Graham, Chris Harbron, Thomas Jaki

**Affiliations:** 1 STOR-i Centre for Doctoral Training, 4396 Lancaster University, Lancaster, UK; 2 Roche Pharmaceuticals, Welwyn Garden City, UK; 3 9147 University of Regensburg, Regensburg, Germany; 4 MRC Biostatistics Unit, 2152 University of Cambridge, Cambridge, UK

**Keywords:** Combination therapies, clinical trials, probability of success, Bayesian, assurance

## Abstract

Combination therapies are becoming increasingly used in a range of therapeutic areas such as oncology and infectious diseases, providing potential benefits such as minimising drug resistance and toxicity. Sets of combination studies may be related, for example, if they have at least one treatment in common and are used in the same indication. In this setting, value can be gained by sharing information between related combination studies. We present a framework that allows the study success probabilities of a set of related combination therapies to be updated based on the outcome of a single combination study. This allows us to incorporate both direct and indirect data on a combination therapy in the decision-making process for future studies. We also provide a robustification that accounts for the fact that the prior assumptions on the correlation structure of the set of combination therapies may be incorrect. We show how this framework can be used in practice and highlight the use of the study success probabilities in the planning of clinical studies.

## 1 Introduction

Combination therapies combine new molecular entities and existing drugs with an aim to produce a synergistic effect whilst also reducing side effects. A synergistic effect is considered to be a positive interaction between the individual components in the combination. Alongside reducing side effects, combination therapies are often able to minimise drug resistance whilst still achieving efficacy.^1^ This is often realised by combining treatments that are similar in terms of the size of their therapeutic effect, but different in terms of their mode of action or toxicity.

There has been a recent rise in the popularity of combination therapies, especially in areas such as oncology. In 2017, there were over 10,000 clinical trials ongoing that contained combinations.^2^ This rise in popularity has also brought with it several new questions and challenges. The question that we will aim to answer in this paper is associated with the potential relationships between the outcomes of combination studies. We may expect the outcome of two combination studies to be related when, for example, they have a particular treatment in common. We look at how we can use the information from related combination studies to inform the probability of success (PoS) of a particular combination study of interest. Typically, a combination therapy will consist of a well-established backbone therapy, such as chemotherapy in oncology, and one or more different add-on treatments. Hence, in this scenario, there would be clear groups of associated combinations, which correspond to the combination therapies that share a backbone treatment.

Therefore, there is much to be gained by considering related combinations. This gain is even more significant when there is little available information on the combination of interest, but a much greater quantity of available information on a related combination, such as the outcome of a Phase III study. This is because of the potential for correlations between the outcomes of related combination studies. Using the additional information from related combinations appropriately may improve the accuracy of the treatment effect estimates, which in turn may lead to improved decision-making in the planning of combination studies through the calculation of the study success probabilities. Improved decision-making may help to reduce the failure rates in the later clinical trial phases or optimise the portfolio.

One of the key estimates that may be used to assist decision-making regarding a potential study is the PoS. Existing methods for calculating the PoS are often based upon the expected power, (Bayesian) predictive power, or assurance. These terms are often used interchangeably in the literature and the assurance will be discussed further and defined in Section 2.1. O’Hagan et al.^3^ presented the concept of the assurance and detailed how it may be used and interpreted. O’Hagan et al.^4^ then provided further discussion of the assurance and how this can be used instead of the power in calculating the required sample size of a study.

The literature on the PoS also covers how this may be used to assist decision-making. Stallard et al.^5^ present an approach that combines Bayesian and frequentist ideas. The decision-making process uses Bayesian methodology in the calculation of the PoS whereas it is assumed that the study design and analysis in Phase III will be frequentist. This approach may be used both at the end of Phase II and at any interim analyses. Sabin et al.^6^ further discussed the use of the PoS in decision-making and presented a two-stage method that starts before Phase II and takes the user through to the end of Phase II decision.

Another area in the literature relevant to the problem that we are interested in relates to the planning of sequences of trials. Whitehead^7^ discusses the problem of designing a series of Phase II studies when the aim is to identify the treatment that should be taken to Phase III. The methodology presented also provides the optimal number of treatments to be tested in Phase II. Existing literature regarding the planning of sequences of trials also includes platform trials^8^ and multi-arm multi-stage trials.^9^ In the setting that we are interested in, however, the related combination studies may not share the same target population or the same indication as is typical in the literature for planning sequences of studies, and the studies might not be available to begin simultaneously.

Bayesian modelling is used in a variety of different areas related to clinical trials such as adaptive designs,^10^ meta-analyses^11^ and assessing safety,^12^ while we present a Bayesian framework that allows the probability distributions of the effect sizes of a group of related combination therapies to be updated based on the outcome of a single combination study. This allows the PoS of related combination studies to be updated. This procedure allows emerging information on related combination therapies to feed into and assist the planning and decision-making process for other potential combination programmes. In line with existing literature, we assume that the design and analysis of the studies are conducted using frequentist methods while the calculation of the PoS will use a Bayesian framework.

In order to provide further motivation and context to this problem, we will consider two historic Phase III trials, and use them to illustrate the methodology throughout the manuscript. We will consider the CLEOPATRA (NCT00567190)^13^ and MARIANNE (NCT01120184)^14^ trials, which both included similar combination therapies in the treatment of patients with HER2-positive breast cancer and used progression-free survival (PFS) as the primary endpoint. The purpose of our method is to capture the relationship between study outcomes, rather than considering the differences between the studies themselves, and to use this to help inform decision-making. Therefore, while the two studies do have several differences, they serve as an example of the type of situation in which the proposed method may be applied.

The CLEOPATRA study is a double-blind study that compared trastuzumab plus docetaxel plus pertuzumab to trastuzumab plus docetaxel plus placebo with a 1:1 allocation ratio.^13^ The MARIANNE study is a multi-arm study that compared trastuzumab emtansine plus placebo and trastuzumab emtansine plus pertuzumab to trastuzumab plus taxane.^14^ The trastuzumab plus taxane arm in the MARIANNE study was open label, whereas the two experimental arms were blinded with respect to pertuzumab or placebo and the allocation ratio was 1:1:1.

Both studies have a control arm that contains trastuzumab and a taxane (the CLEOPATRA study also contains placebo) and an experimental arm that includes pertuzumab. We will therefore aim to use the CLEOPATRA study to draw inference upon the outcome of a modified hypothetical two-arm version of the MARIANNE study that compares trastuzumab plus taxane to trastuzumab emtansine plus pertuzumab; we will not consider the trastuzumab emtansine plus placebo arm. From now on, we will refer to this hypothetical study as the modified MARIANNE (mod-MARIANNE) study.

It is clear that there are several differences between the two studies, such as the blinding and the number of arms. Irrespective of this, we believe that the outcome of one study could be informative for the other and hence these studies will be used for illustration of the methodology.

In Section 2, we present the framework and methodology for updating the PoS of related combination studies. We also provide an extension that accounts for the fact that the treatment effects, hence study success probabilities, of the combinations may not be correlated. In Section 3, we present the results of a simulation study. We provide a discussion of the approach in Section 4.

## 2 Methods

In this section, we build the framework that allows the PoS of a combination study to be updated based on the outcome of a related combination study with the aim to assist decision-making.

First, we update the distributions of related combination therapies based on the outcome of a single combination study using a Bayesian framework. Then, using the updated marginal distributions, we can find the PoS for all remaining studies. We also provide an extension that allows us to consider the fact that the assumption of related combination studies being positively correlated might not always hold and account for this in our PoS calculations.

### 2.1 Framework

For illustrative purposes, let us first consider a pair of related combinations, for example A+B and A+C , which we might be comparing to a similar control treatment, before extending the problem to a set of n related combinations. We will refer to combinations as ‘related’ when they have at least one monotherapy in common and there is reason to believe that the performance of the combinations will be related. An example of this might be in oncology where A is a backbone treatment, such as chemotherapy, and B and C are potential add-on treatments with different modes of action.

We are interested in calculating the PoS for one combination study based on the study results of a related combination. In order to calculate the PoS we will follow the method presented by O’Hagan et al.^4^ to calculate the assurance, which is defined by

(1) PoS=∫P(studysuccess∣θ)P(θ∣data)dθ

where θ represents the treatment effect. We can often find a closed-form solution for the assurance. For example, in the case of a two-sided superiority trial with normally distributed outcomes and known variance, the assurance for rejecting the null hypothesis of no treatment difference in favour of the experimental treatment is given by

(2) $$\mathrm{PoS}=1-\Phi \left(\frac{V^{-0.5}Z_{\alpha /2}-\mu }{\sqrt{V^{-1}+\sigma^{2}}}\right)$$

where $V^{-1}$ is the sampling variance of the planned study, α is the significance level and μ and $\sigma^{2}$ are the mean and variance of the distribution representing our beliefs on θ , respectively, and might be based on historical data.^4^ When a closed-form solution for the assurance is not available, Bayesian clinical trial simulation can be used to estimate it.^4^ Wang et al.^15^ also demonstrate how the assurance may be calculated using Bayesian modelling and trial simulation. Alternative distribution-based definitions of the PoS could also be used in our presented framework, such as the Bayesian PoS presented by Ibrahim et al.^16^ or the extensions of the Bayesian expected power presented by Liu.^17^

In order to calculate the PoS and update it using related combination study data, we need to consider the treatment effects of the combinations of interest. For the simple example of combinations A+B and A+C , we will use $\theta_{1}$ and $\theta_{2}$ to represent the treatment effects of A+B and A+C , respectively. We will assume that $\theta_{1}$ and $\theta_{2}$ are measured on the same scale and are therefore directly comparable.

Before a clinical trial begins, the study team will have some idea as to how the therapy may be expected to perform based on historical data and expert opinion. In order to capture these beliefs we can specify a prior distribution on the parameter of interest. This prior distribution is able to capture the expected value of the treatment effect and also the level of uncertainty in this value. There is extensive literature on prior elicitation in the setting of a clinical trial, with one of the most commonly discussed methods being the SHELF framework.^18^

We will represent the prior beliefs for the treatment effects of the two combination therapies, $\theta =(\theta_{1},\theta_{2}{)}^{T}$ , by the multivariate normal (MVN) distribution. We can write this as θ∼MVN(μ,Σ) or, alternatively for the two combination example,

$$\left(\begin{array}{c} \theta_{1}\\ \theta_{2} \end{array}\right)∼\mathrm{MVN}\left(\left(\begin{array}{c} \mu_{1}\\ \mu_{2} \end{array}\right),\left(\begin{array}{cc} \sigma_{1}^{2} & \rho_{12}\sigma_{1}\sigma_{2}\\ \rho_{12}\sigma_{1}\sigma_{2} & \sigma_{2}^{2} \end{array}\right)\right)$$

where $\mu_{i}$ and $\sigma_{i}^{2}$ represent the prior expectation and prior variance for $\theta_{i}$ . In this model, the parameter $\rho_{ij}$ will be used to define the level of borrowing across the two combinations. The reasons why we can use the interpretation of $\rho_{ij}$ as the degree of borrowing will be discussed in Section 2.3.

When determining an appropriate value for $\rho_{12}$ , one could consider a thought experiment using studies relating to $\theta_{1}$ and $\theta_{2}$ . For example, if these relate to the combinations of A+B and A+C then one might consider either the outcome of these combinations in different indications or alternatively the outcomes of B and C when paired with different backbone treatments. If these outcomes are typically positive or negative simultaneously then a higher value of $\rho_{12}$ may be appropriate. If there was little or no pattern between the pairs then a lower value of $\rho_{12}$ would be more appropriate.

It should be noted that this model does not aim to capture synergism or antagonism within the components of the combinations, instead, it aims to capture similarities across the combinations, which will allow us to learn across the combinations.

In the case where there are n related combinations, we would specify the prior beliefs using

$$\left(\begin{array}{c} \theta_{1}\\ \theta_{2}\\ \vdots \\ \theta_{n} \end{array}\right)∼\mathrm{MVN}\left(\left(\begin{array}{c} \mu_{1}\\ \mu_{2}\\ \vdots \\ \mu_{n} \end{array}\right),\left(\begin{array}{cccc} \sigma_{1}^{2} & \rho_{12}\sigma_{1}\sigma_{2} & \cdots & \rho_{1n}\sigma_{1}\sigma_{n}\\ \rho_{12}\sigma_{1}\sigma_{2} & \sigma_{2}^{2} & \cdots & \rho_{2n}\sigma_{2}\sigma_{n}\\ \vdots & \vdots & \ddots & \vdots \\ \rho_{1n}\sigma_{1}\sigma_{n} & \rho_{2n}\sigma_{2}\sigma_{n} & \cdots & \sigma_{n}^{2} \end{array}\right)\right).$$

This is the distribution that we will update based on the outcome of a combination study relating to one of the $\theta_{i}$ variables. We will then use the updated distribution to calculate the PoS for future combination studies.

In order to specify the prior distribution for our illustrative example we will let $\theta_{M}$ be the treatment difference for the mod-MARIANNE study comparing trastuzumab plus taxane with trastuzumab emtansine plus pertuzumab. We will further let $\theta_{C}$ be the treatment difference on the log hazard ratio (HR) scale for the CLEOPATRA study comparing trastuzumab plus docetaxel plus placebo with trastuzumab plus docetaxel plus pertuzumab.

We will specify the prior means of both $\theta_{M}$ and $\theta_{C}$ to be equivalent to a HR of 0.75, $\mu_{M}=\mu_{C}=-\log\;(0.75)$ , which is equal to the reference value that was used to power both of the studies. We will specify a prior correlation of $\rho_{MC}=0.6$ to reflect the belief that the outcomes of the studies are related along with our interest in using the outcome of one of the studies to inform our beliefs about the other. If the two studies only differed in one aspect but were otherwise identical, we may consider using a higher correlation. However, since the studies differ in several ways, we have decided to use a lower correlation to reflect the uncertainty caused by the differences. Finally, we will specify a prior variance of 0.08 on both treatment effects. This is equivalent to the posterior variance after observing approximately 50 PFS events, given an uninformative prior variance. This will give a bivariate prior of

$$\left(\begin{array}{c} \theta_{M}\\ \theta_{C} \end{array}\right)∼\mathrm{MVN}\left(\left(\begin{array}{c} 0.288\\ 0.288 \end{array}\right),\left(\begin{array}{cc} 0.08 & 0.05\\ 0.05 & 0.08 \end{array}\right)\right).$$

### 2.2 Score statistics

As we observe further clinical studies on the combinations, we want to update this distribution to reflect the information gained from these new studies. We assume that these studies will be designed and analysed using frequentist methodology using a test based upon a likelihood function. Therefore, to summarise the outcome of study i , we will use the score statistic, $Z_{i}$ , and the Fisher information, $V_{i}$ , of the test with null hypothesis $\theta_{i}=0$ .^19^

The score statistic can be considered as a measure of the benefit of the experimental treatment, based on what was observed in the study, and the Fisher information is a measure of how much information on $\theta_{i}$ is contained in $Z_{i}$ . We will denote the maximum likelihood estimate of $\theta_{i}$ by $\hat{\theta_{i}}$ . When the study sample size is large and $\theta_{i}$ is small, the score statistic is approximately normally distributed with mean given by $V_{i}\theta_{i}$ and variance given by $V_{i}$ where $\theta_{i}$ is the true value of the treatment effect and ${\hat{\theta }}_{i}\approx Z_{i}/V_{i}$ .^19^ Therefore,

$${\hat{\theta }}_{i}\;\;\dot{∼}\;\;N\left(\theta_{i},V_{i}^{-1}\right).$$

Note that this normal approximation holds for many endpoints, which is one of the main reasons that we consider the score statistic in our framework.

If we only consider the marginal prior distribution of $\theta_{i}$ , then, since the normal distribution is a conjugate prior for normally distributed data, we could find the posterior distribution of $\theta_{i}|Z_{i}$ , or $\theta_{i}|{\hat{\theta }}_{i}$ and this would also follow the univariate normal distribution.

In our setting, however, we consider these parameters in a vector represented by θ and we do not observe all dimensions of θ simultaneously, but observe the outcome of one combination study at a time. Therefore, the distribution of the score statistic, $Z_{i}$ , will remain one-dimensional. However, we will still want to update the distribution of θ each time we observe new data.

In our illustrative example, the CLEOPATRA study was the first of the two studies to be conducted therefore we will use the information from the CLEOPATRA study to update our beliefs about the PoS of the mod-MARIANNE study. The CLEOPATRA study observed 604 PFS events, 320 in the control arm and 284 in the experimental arm, and the observed HR was 0.68.^20^ We are able to find $Z_{C}$ and $V_{C}$ for the study using

(3) $$V\approx e\times R/(R+1{)}^{2}\;\mathrm{and}\;Z=-V\log\;(\mathrm{HR})$$

where e is the number of PFS events and R:1 is the allocation ratio.^19^ This gives $V_{C}=151$ and $Z_{C}=58.235$ .

### 2.3 Method

In this section, we will consider the case where there are n combinations of interest and we observe the outcomes of m studies simultaneously and wish to update the probability distribution for the n combinations based on these results. We will illustrate the method using our earlier example containing two combinations, n=2 , where we observe results on one of these combinations, m=1 .

Let Z be the vector of score statistics for the m observations and let V be the associated diagonal matrix of Fisher information,

$$Z=\left(\begin{array}{c} Z_{1}\\ Z_{2}\\ \vdots \\ Z_{m} \end{array}\right)\;\mathrm{and}\;V=\left(\begin{array}{cccc} V_{1} & 0 & \cdots & 0\\ 0 & V_{2} & \cdots & 0\\ \vdots & \vdots & \ddots & \vdots \\ 0 & 0 & \cdots & V_{m} \end{array}\right)$$

where we assume that θ is ordered such that we observe outcomes on the first m components. Then, we can write $\hat{\theta }=V^{-1}Z=({\hat{\theta }}_{1},\ldots ,{\hat{\theta }}_{m}{)}^{T}.$

In order to consider the distribution of $\hat{\theta }|\theta$ , we will also introduce a matrix, A of dimension m×n , which selects the components of θ that were observed and are included in $\hat{\theta }$ . When θ is ordered such that we observe outcomes on the first m components, the components of this matrix would be given by

$$A_{ii}=1\;\;\mathrm{for}\;i=1,\ldots ,m\;\mathrm{and}\;A_{ij}=0\;\;\forall \;\;i\neq j.$$

We may then write

$$\hat{\theta }|\theta \;\;\dot{∼}\;\;\mathrm{MVN}\left(A\theta ,V^{-1}\right).$$

We can then find the posterior distribution of $\theta |\hat{\theta }$ as follows.^21^

$$\begin{array}{rl} \;p\left(\theta |\hat{\theta }\right) & \propto p\left(\hat{\theta }|\theta \right)p\left(\theta \right)\\ & \propto \exp\;\left\{-\frac{1}{2}\left[{\left(\hat{\theta }-A\theta \right)}^{T}V\left(\hat{\theta }-A\theta \right)\right]\right\}\times \exp\;\left\{-\frac{1}{2}\left[{\left(\theta -\mu \right)}^{T}\Sigma^{-1}\left(\theta -\mu \right)\right]\right\}\\ & \propto \exp\;\left\{-\frac{1}{2}\left[\theta^{T}\left(A^{T}VA+\Sigma^{-1}\right)\theta -2\theta^{T}\left(A^{T}V\hat{\theta }+\Sigma^{-1}\mu \right)\right]\right\} \end{array}$$

Therefore,

$$\theta |\hat{\theta }\;\;\dot{∼}\;\;\mathrm{MVN}\left({\left(\Sigma^{-1}+A^{T}VA\right)}^{-1}\left(\Sigma^{-1}\mu +A^{T}V\hat{\theta }\right),{\left(\Sigma^{-1}+A^{T}VA\right)}^{-1}\right).$$

For our simple example containing combinations A+B and A+C , this gives

$$\left(\begin{array}{c} \theta_{1}\\ \theta_{2} \end{array}\right)|Z_{2}=z_{2}∼\mathrm{MVN}\left(\left(\begin{array}{c} \mu_{1}-\frac{\rho_{12}\sigma_{1}\sigma_{2}V_{2}}{1+V_{2}\sigma_{2}^{2}}\mu_{2}+\frac{\rho_{12}\sigma_{1}\sigma_{2}}{1+V_{2}\sigma_{2}^{2}}z_{2}\\ \frac{1}{1+V_{2}\sigma_{2}^{2}}\mu_{2}+\frac{\sigma_{2}^{2}}{1+V_{2}\sigma_{2}^{2}}z_{2} \end{array}\right),\left(\begin{array}{cc} \sigma_{1}^{2}-\frac{V_{2}\rho_{12}^{2}\sigma_{1}^{2}\sigma_{2}^{2}}{1+V_{2}\sigma_{2}^{2}} & \frac{\rho_{12}\sigma_{1}\sigma_{2}}{1+V_{2}\sigma_{2}^{2}}\\ \frac{\rho_{12}\sigma_{1}\sigma_{2}}{1+V_{2}\sigma_{2}^{2}} & \frac{\sigma_{2}^{2}}{1+V_{2}\sigma_{2}^{2}} \end{array}\right)\right).$$

Here we see that the parameter $\rho_{12}$ defines how far and in which direction the mean of $\theta_{1}$ shifts from its prior mean. If $\rho_{12}$ is positive and $Z_{2}/V_{2}>\mu_{2}$ then the posterior mean for $\theta_{1}$ will be greater than the prior mean, $\mu_{1}$ . This represents the assumption that if we specify $\rho_{12}>0$ , which means that we specify that $\theta_{1}$ and $\theta_{2}$ are correlated, then our prior beliefs would also be correlated. Therefore, if we observe an outcome on $\theta_{2}$ that suggests that our prior mean was an underestimate of the truth, $Z_{2}/V_{2}>\mu_{2}$ , then we would probably also believe that $\mu_{1}$ is also an underestimate of the truth hence the mean of $\theta_{1}$ should also be increased. Similarly, if $Z_{2}/V_{2}<\mu_{2}$ , including the case where there is no effect observed, then the posterior mean will decrease from the prior mean. The amount by which the mean will shift will also be dependent on our prior variance and observed variance and on the value of $\rho_{12}$ , which we specify in advance. Consequently, when specifying $\rho_{12}$ , we should consider this as a measure of how far we would want our unobserved treatment effect mean to shift based on indirect data.

Note that interestingly we can also use the Kalman Filter^22^ and Gaussian Markov Random Fields^23^ to tackle the problem presented here, which lead to the same posterior distribution. Details of this can be found in Appendix A.

We can then find the updated PoS for a study on A+B using equation (1), where P(θ|data) corresponds to the marginal distribution for $\theta_{1}$ , or alternatively we could use Bayesian clinical trial simulation to estimate this expression. Note that, in order to calculate this value, we will also require the definition of study success for the study of A+B .

Following the above approach, the posterior distribution for our illustrative example is given by

$$\left(\begin{array}{c} \theta_{M}\\ \theta_{C} \end{array}\right)|Z_{C}=58.235∼\mathrm{MVN}\left(\left(\begin{array}{c} 0.342\\ 0.378 \end{array}\right),\left(\begin{array}{cc} 0.053 & 0.004\\ 0.004 & 0.006 \end{array}\right)\right).$$

We can find the PoS of the mod-MARIANNE study using equation (1) along with this posterior distribution and information on the study design. We will use a significance level of α=0.05 for the mod-MARIANNE study, which was also used in the MARIANNE study although it was split between the two comparisons. A power of 80% and a target HR of 0.75 will be used, as in the MARIANNE study design. This results in

$$\begin{array}{rl} V_{M} & \approx {\left(\frac{Z_{0.05/2}+Z_{1-0.8}}{-\log\;\left(0.75\right)}\right)}^{2}\\ & =94.838 \end{array}$$

following the method presented by Whitehead.^19^ This can be found using equation (3) and replacing e by the sample size formula for survival endpoints. Hence, the PoS of the mod-MARIANNE study, based on the results of the CLEOPATRA study, is 0.711 . If we had not included the information from the CLEOPATRA study, the PoS based on the marginal prior distribution would have been 0.613.

If we use a prior correlation of 0.4, instead of 0.6 as above, then we would have a posterior distribution of

$$\left(\begin{array}{c} \theta_{M}\\ \theta_{C} \end{array}\right)|Z_{C}=58.235∼\mathrm{MVN}\left(\left(\begin{array}{c} 0.324\\ 0.378 \end{array}\right),\left(\begin{array}{cc} 0.068 & 0.002\\ 0.002 & 0.006 \end{array}\right)\right)$$

which would lead to a PoS of 0.669 for the mod-MARIANNE study. These results are more conservative as we are choosing to borrow less information from the CLEOPATRA study, but the posterior PoS is still increased compared to the prior PoS. Alternatively, the posterior distribution based on a prior correlation of 0.8 results in a PoS of 0.777 for the mod-MARIANNE study. This illustrates the effect that the prior correlation has on the inference we make based on the output of this approach.

The three-arm MARIANNE study^14^ was completed with study parameters as described previously and α=0.05 split between the two comparisons of the experimental treatments with a control. The results of the study showed both experimental arms to be non-inferior, but not superior, to the control arm in terms of PFS. The stratified HR for PFS for trastuzumab emtansine plus pertuzumab vs trastuzumab plus taxane was 0.87.^14^ It is noted by Perez et al.^14^ that the median PFS of the control arm that was assumed when designing the study was shorter than what was observed. The median PFS of the control arm was assumed to be 11 months, which was based on information that was available at the time. The median PFS observed in the study control group was 13.7 months, which is similar to the estimate from more recent studies.^14^

Note that, in this illustrative example, there were several differences between the two studies, yet our method is still able to add benefit in this case. This is because our method allows the user to consider how the beliefs regarding a treatment effect change based on related study outcomes and the effect that this has on the probability of study success. The method does not require a high level of correlation between the treatment effects, nor does it require specific information on the similarities between the studies, it simply requires a parameter for the level of borrowing across the studies. This means that it may be applicable in a wide range of settings and may also be used to help inform and assist decision-making.

If there is doubt regarding the relationship between the study outcomes, the user might prefer the amount of borrowing to be dependent on the observed data. This would allow for a small amount of borrowing when the observed data suggests little correlation between study outcomes and a higher level of borrowing when the data suggests a relationship between outcomes. We present a robustification in the next section that aims to capture this requirement.

### 2.4 Robustification

In Section 2.3, we outlined the method that can be used to update the distribution of a set of related combination therapies based on the outcome of a single combination study. Updating a distribution given relevant observations allows us to improve the accuracy of our estimates. However, thus far, we have assumed that all of the therapies in our set of ‘related’ combinations are truly correlated and that there is something to be gained from sharing information across the different combinations, but this might not always be the case.

In this section, we will consider an extension to the method that allows us to take into account the fact that the outcomes of two ‘related’ combination studies may not actually be correlated, despite initial beliefs, and robustify our procedure against this. Since we are only observing the outcome of one combination study at a time, we do not have the opportunity to learn from pairs of outcomes. Therefore, we cannot learn about the correlation and hence update our model using this. Instead, we will consider how emerging data aligns with our prior beliefs, which is similar to recent work on extrapolation.^24^

If we observe a study for combination i , which we summarise using $Z_{i}$ and $V_{i}$ , we would want to update our beliefs about $\theta_{i}$ using the study data. However, we may not necessarily want to update our beliefs about $\theta_{j}$ , for i≠j . When the posterior expectation of $\theta_{j}$ given $Z_{i}$ is similar to our prior expectation of $\theta_{j}$ , we might wish to include this additional information, as it does not seem too controversial given what we believed initially. However, if the marginal posterior of $\theta_{j}$ is shifted by a much greater magnitude in the location given $Z_{i}$ , this may cause some concern as to whether or not we are comfortable including this indirect information and a study team may wish to be more conservative in this case. Therefore, our extension will allow us to include less indirect information when the shift is large.

First, we will consider a mixture prior on θ made up of two distributions. In the first distribution, the correlation between combinations will be set equal to zero, which implies no borrowing across combinations, and in the second distribution, the correlation will be set to the level that we would choose if we knew that they were in fact correlated. This value, as before, may be thought of as the amount that we would like to borrow across the combinations. We will write this mixture prior as

$$\left(\begin{array}{c} \theta_{1}\\ \theta_{2} \end{array}\right)∼\omega_{0}^{0}\times \mathrm{MVN}\left(\left(\begin{array}{c} \mu_{1}\\ \mu_{2} \end{array}\right),\left(\begin{array}{cc} \sigma_{1}^{2} & 0\\ 0 & \sigma_{2}^{2} \end{array}\right)\right)+\omega_{1}^{0}\times \mathrm{MVN}\left(\left(\begin{array}{c} \mu_{1}\\ \mu_{2} \end{array}\right),\left(\begin{array}{cc} \sigma_{1}^{2} & \rho_{12}\sigma_{1}\sigma_{2}\\ \rho_{12}\sigma_{1}\sigma_{2} & \sigma_{2}^{2} \end{array}\right)\right)$$

where the weights $\omega_{0}^{0}$ and $\omega_{1}^{0}$ may be thought of as the prior probabilities that $\theta_{1}$ and $\theta_{2}$ are uncorrelated and correlated, respectively, and $\omega_{0}^{0}$ + $\omega_{1}^{0}=1$ .

If we were to update this mixture in the standard way then the weights would remain unchanged despite the gain in information. Therefore, we will develop some further methodology in order to update the weights and use the methodology from Section 2.3 to update the separate components.

Let us first consider the properties that we will want this procedure to have. Firstly, we want it to consider the amount that the distribution has shifted and to assign a higher weight to the uncorrelated distribution if this shift is too large, i.e. moves ‘too far’ from what we initially thought was realistic. Conversely, if the shift in the marginal posterior mean is small and the study size is large, we would want to assign a higher weight to the correlated distribution. Furthermore, if the observed study is small, then we only want the weights to shift a small amount compared to how much they would have shifted given equivalent results from a large study.

We want to update the weights by combining the prior weights, $\omega_{0}^{0}$ and $\omega_{1}^{0}$ , with some new information that we will contain in a yet-to-be-defined measure, p . This value will be used to quantify how much of the new information we want to borrow. The posterior weights will be given by

(4) $$\omega_{0}^{1}=\frac{(1-p)\omega_{0}^{0}}{(1-p)\omega_{0}^{0}+p\omega_{1}^{0}}\;\mathrm{and}\;\omega_{1}^{1}=\frac{\;p\omega_{1}^{0}}{(1-p)\omega_{0}^{0}+p\omega_{1}^{0}}.$$

We will consider two ways of specifying p : a hypothetical posterior approach and a limiting posterior approach. Both of these approaches have desirable properties that align with the requirements we outlined above for the weighting procedure.

For the hypothetical posterior approach, we construct a hypothetical normally distributed posterior for $\theta_{1}$ given $Z_{2}$ that has a posterior mean equal to the prior mean, $\mu_{1}$ , and posterior variance equal to the posterior variance found doing the usual update given $V_{2}$ . Hence, the hypothetical posterior is given by

$$N\left(\mu_{1},\sigma_{1}^{2}-\frac{V_{2}\rho_{12}^{2}\sigma_{1}^{2}\sigma_{2}^{2}}{1+V_{2}\sigma_{2}^{2}}\right).$$

For the limiting posterior approach, we construct the limiting posterior distribution for $\theta_{1}$ given $Z_{2}$ as $V_{2}\to \infty$ that has a posterior mean equal to the prior mean. Hence, here the posterior mean will be given, as before, by $\mu_{1}$ , and the posterior variance will be given by $\sigma_{1}^{2}(1-\rho_{12}^{2})$ so that the limiting posterior is given by

$$N\left(\mu_{1},\sigma_{1}^{2}\left(1-\rho_{12}^{2}\right)\right).$$

Our interest, however, lies in the location of the mean. Therefore, we will consider the lower and upper quartiles of these distributions, which we will denote by $\left[\theta_{1,l}^{H},\theta_{1,u}^{H}\right]$ and $\left[\theta_{1,l}^{L},\theta_{1,u}^{L}\right]$ for the hypothetical and limiting posterior distributions, respectively.

We then want to compare these quartiles with the posterior that we find using the original procedure given the observed value of $Z_{2}$ and $V_{2}$ . In order to do this we will truncate the posterior at its upper and lower quartiles, $\theta_{1,l}$ and $\theta_{1,u}$ .

$$\theta_{1}|Z_{2}=z_{2}∼TN\left(\mu_{1}-\frac{\rho_{12}\sigma_{1}\sigma_{2}V_{2}}{1+V_{2}\sigma_{2}^{2}}\mu_{2}+\frac{\rho_{12}\sigma_{1}\sigma_{2}}{1+V_{2}\sigma_{2}^{2}}z_{2},\sigma_{1}^{2}-\frac{V_{2}\rho_{12}^{2}\sigma_{1}^{2}\sigma_{2}^{2}}{1+V_{2}\sigma_{2}^{2}},\theta_{1,l},\theta_{1,u}\right)$$

Then, we will take the value of p , the value that we use to update the weights, to be

$$p=P\left(\theta_{1}\in \left[\theta_{1,l}^{q},\theta_{1,u}^{q}\right]|Z_{2}=z_{2}\right)$$

using the truncated posterior distribution of $\theta_{1}$ where q=H,L represents the hypothetical or limiting posterior distributions. This value is the probability of the truncated posterior distribution lying within the lower and upper quartiles of the hypothetical/limiting posterior distributions. Hence, when the shift is small, this probability will be large as there will be a large overlap between the distributions. On the other hand, when the shift is large, this probability will be small, especially since we are taking the posterior truncated at the lower and upper quartiles. Note that if the posterior is perfectly aligned with the hypothetical or limiting posterior distribution then p will take a value of 1.

**Figure 1.**
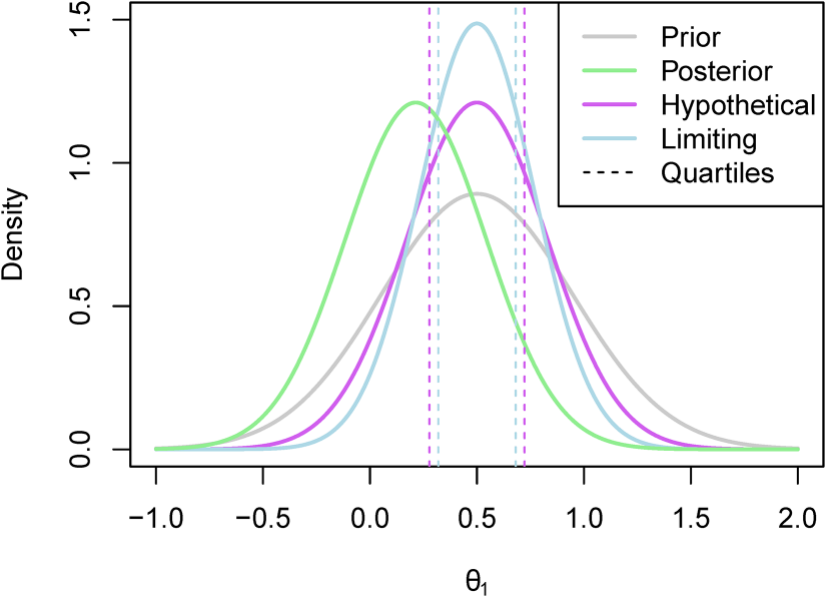
The hypothetical and limiting posterior distributions for an illustrative example.

An example of what the hypothetical and limiting posterior distributions may look like can be found in Figure 1. In this example, our posterior beliefs do not align with our prior beliefs as we see a shift in the mean. However, there still seems to be quite a large amount of overlap between the posterior distribution and the hypothetical posterior distribution, while there is less so with the limiting posterior distribution. This figure also serves to illustrate why we consider the truncated posterior rather than the original posterior. Recall that the posterior distribution here is based only on ‘related’ data, and not on direct data. Therefore, considering the truncated distribution allows us to reduce the overlap in cases such as this one where the means are far enough apart for us to consider it to be a potential reason not to borrow from the ‘related’ combination.

Once we have found our chosen value of p , we are able to find the updated weights using equation (4) and then our mixture posterior will be given by

$$\begin{array}{rl} \left(\begin{array}{c} \theta_{1}\\ \theta_{2} \end{array}\right)|Z_{2} & =z_{2}∼\omega_{0}^{1}\times \mathrm{MVN}\left(\left(\begin{array}{c} \mu_{1}\\ \frac{1}{1+V_{2}\sigma_{2}^{2}}\mu_{2}+\frac{\sigma_{2}^{2}}{1+V_{2}\sigma_{2}^{2}}z_{2} \end{array}\right),\left(\begin{array}{cc} \sigma_{1}^{2} & 0\\ 0 & \frac{\sigma_{2}^{2}}{1+V_{2}\sigma_{2}^{2}} \end{array}\right)\right)\\ & \;+\omega_{1}^{1}\times \mathrm{MVN}\left(\left(\begin{array}{c} \mu_{1}-\frac{\rho_{12}\sigma_{1}\sigma_{2}V_{2}}{1+V_{2}\sigma_{2}^{2}}\mu_{2}+\frac{\rho_{12}\sigma_{1}\sigma_{2}}{1+V_{2}\sigma_{2}^{2}}z_{2}\\ \frac{1}{1+V_{2}\sigma_{2}^{2}}\mu_{2}+\frac{\sigma_{2}^{2}}{1+V_{2}\sigma_{2}^{2}}z_{2} \end{array}\right),\left(\begin{array}{cc} \sigma_{1}^{2}-\frac{V_{2}\rho_{12}^{2}\sigma_{1}^{2}\sigma_{2}^{2}}{1+V_{2}\sigma_{2}^{2}} & \frac{\rho_{12}\sigma_{1}\sigma_{2}}{1+V_{2}\sigma_{2}^{2}}\\ \frac{\rho_{12}\sigma_{1}\sigma_{2}}{1+V_{2}\sigma_{2}^{2}} & \frac{\sigma_{2}^{2}}{1+V_{2}\sigma_{2}^{2}} \end{array}\right)\right). \end{array}$$

If we decided to use the standard mixture approach, where the posterior weights are not updated, then we could also use the above distribution, but we would have $\omega_{0}^{1}=\omega_{0}^{0}$ and $\omega_{1}^{1}=\omega_{1}^{0}$ , rather than the weights given in equation (4).

Following the same approach outlined in Section 2.3, this posterior can be used to calculate the success probability of a combination study of interest by using the assurance as presented by O’Hagan et al.^4^

If we applied this approach to our illustrative example then we would have a posterior distribution of

$$\begin{array}{rl} \left(\begin{array}{c} \theta_{M}\\ \theta_{C} \end{array}\right)|Z_{C} & =58.235∼\omega_{0}^{1}\times \mathrm{MVN}\left(\left(\begin{array}{c} 0.288\\ 0.378 \end{array}\right),\left(\begin{array}{cc} 0.08 & 0\\ 0 & 0.006 \end{array}\right)\right)\\ & \;+\omega_{1}^{1}\times \mathrm{MVN}\left(\left(\begin{array}{c} 0.342\\ 0.378 \end{array}\right),\left(\begin{array}{cc} 0.053 & 0.004\\ 0.004 & 0.006 \end{array}\right)\right) \end{array}$$

where the values of $Z_{C}$ , $V_{C}$ , μ and Σ were given in Section 2.3.

**Figure 2.**
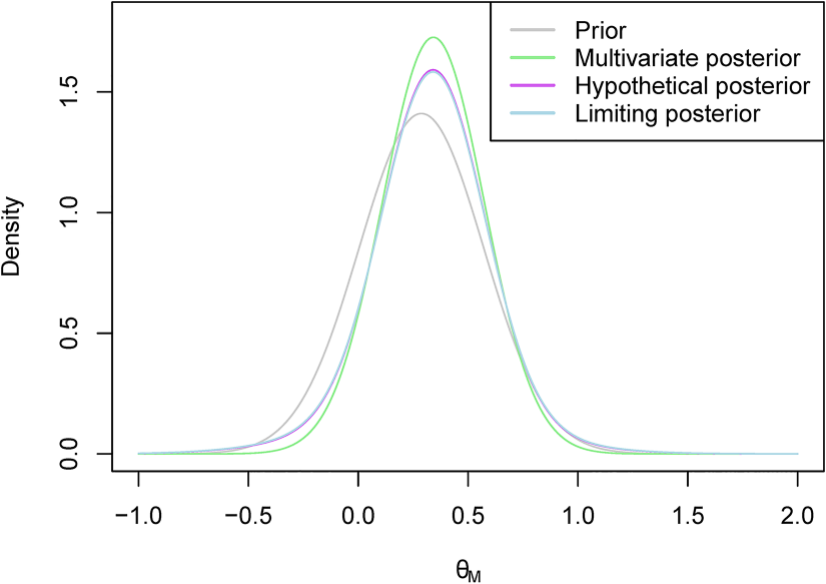
The marginal posterior distributions of $\theta_{M}$ for the illustrative example.

If we set $\omega_{0}^{0}=0.5$ and $\omega_{1}^{0}=0.5$ , then the hypothetical posterior approach would lead to $\omega_{0}^{1}=0.16$ and $\omega_{1}^{1}=0.84$ , which would give a PoS of the mod-MARIANNE study of 0.689. The limiting posterior approach yields similar results with $\omega_{0}^{1}=0.17$ and $\omega_{1}^{1}=0.83$ and a PoS of 0.688. As we would expect, the PoS under the robustified approach is between the PoS from the marginal prior of $\theta_{M}$ , 0.613, and the standard multivariate procedure, 0.711. They are also higher than the PoS when the prior correlation was set to 0.4, but this would not necessarily be the case if the observed data were further away from our prior beliefs. Figure 2 shows the different marginal posterior distributions of $\theta_{M}$ for this illustrative example.

If we wished to use this extension for more than two combinations, we would simply need to split our vector of random variables, θ , into pairs of random variables, $(\theta_{i},\theta_{j})\;\forall \;i\neq j$ . Each pair would need to contain $\theta_{i}$ , the treatment effect that we will observe some data for, alongside one of the correlated treatment effects. This would allow us to find the values of p in the same way presented here and would account for the fact that some pairs of $\theta_{i}$ and $\theta_{j}$ might be strongly correlated, which would lead to a high weight on the correlated component, whereas other pairs may be uncorrelated, which would lead to a high weight on the uncorrelated component of the mixture. Thus, splitting the full n -dimensional problem into n−1 two-dimensional problems may be a more appropriate approach in this setting.

## 3 Results

In this section, we will illustrate the performance of these methods by looking at the posterior distributions and the success probabilities that these methods lead to in a simulation study. We will compare the results of the proposed multivariate methods to the results of only marginal updating i.e. the univariate alternative. We will also include the standard mixture approach as mentioned in Section 2.4, which is the approach where the weights are not updated, in the simulation study for comparison to the hypothetical and limiting posterior mixture approaches. We will use the assurance to calculate the study success probabilities in the simulation study, as in previous sections, but it should be noted that other methods for calculating the PoS may also be used.

In order to provide a complete picture of the way these multivariate methods perform compared to the univariate alternative, we will consider different sets of prior distributions that may have arisen from historical data such as the results of a small study. We will take the true value of $\theta_{1}$ and $\theta_{2}$ to be equal to 0.5.

We will assume that the prior information on both of these parameters is equivalent to having a prior variance of 0.2. This is approximately equal to having an uninformative ‘pre-prior’ and updating based on the outcome of a study involving 20 patients with normally distributed responses.

We will assume that we observe the outcome of a study on A+C and want to update the distributions for both A+B and A+C based on this. If we do not consider borrowing information across the combinations, the prior distribution will represent all of the information, or beliefs, that we have regarding A+B and we will make our decisions based on this distribution in the univariate setting.

### 3.1 Effect of the sample size on the PoS

**Figure 3.**
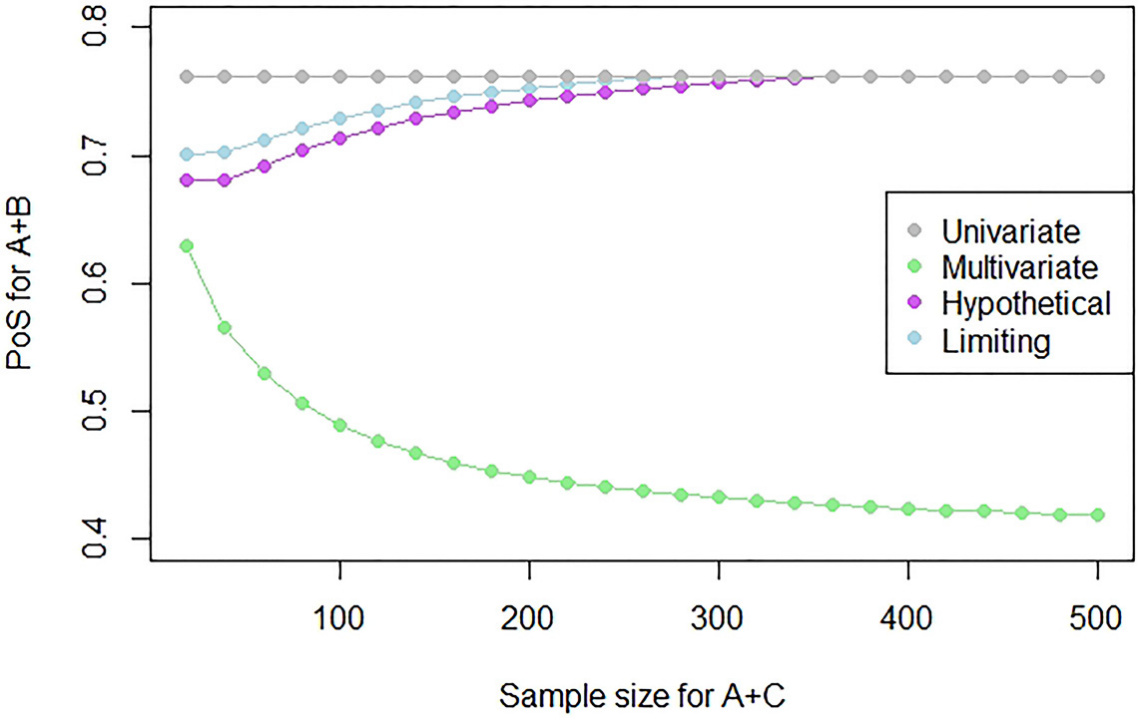
Plot showing the PoS for a study on $\theta_{1}$ as the sample size of a study on $\theta_{2}$ increases, which has an outcome of $Z_{2}/V_{2}=0$ .

In order to illustrate what might happen to the PoS for different sample sizes, we can consider a fixed study outcome and find the PoS using this outcome with different sample sizes. Figure 3 shows how the PoS for a study on A+B changes as the sample size changes in the observed study on A+C for a fixed outcome of no effect, $Z_{2}/V_{2}=0$ , and a prior mean of $\mu =(0.5,0.5{)}^{T}$ . We define the PoS to be the assurance for a future two-sided superiority study on A+B that has a planned sample size of 500 and a significance level of 0.05.

We see that as the sample size increases, the PoS of the multivariate approach decreases as there is more evidence to suggest that $\theta_{2}=0$ , which would suggest that, if $\theta_{1}$ and $\theta_{2}$ are correlated, then our prior mean for $\theta_{1}$ is also an overestimate. However, the hypothetical and limiting posterior approaches originally have a lower PoS than the univariate method, but as the sample size increases, so will the shift from the prior to the posterior mean of $\theta_{1}$ . Since these approaches will assign a higher weight to the univariate approach when the shift size increases, the success probabilities from the hypothetical and limiting posterior approaches tend towards the PoS of the univariate approach as the sample size increases. Note that no method here is performing better than the other, as we have not defined what the truth is and our prior mean for $\theta_{1}$ might be an overestimate, or $\theta_{1}$ and $\theta_{2}$ might be uncorrelated. This figure simply serves as an illustration of how the different methods assign the PoS.

### 3.2 Simulation set-up

We will consider the sample size of the study of A + C to be equal to 500 as we would be most interested in borrowing information and using this methodology when we observe the outcome of a relatively large (e.g. Phase III) study.

In order to account for the variability in the treatment effect estimate that we would have based on a small prior study, we will consider three different prior means for $\theta_{2}$ . Results for different prior means of $\theta_{1}$ may be found in Appendix B. We will consider prior means of 0.2, 0.5 and 0.8 for $\theta_{2}$ . These values correspond to the means we would find given an uninformative ‘pre-prior’ and an update based on the quartiles of the distribution of the score statistic when the true value of $\theta_{i}$ is equal to 0.5 and the value of $V_{i}$ is equivalent to a study size of 20 patients with normally distributed responses.

We set up the different prior distributions and we simulate 10,000 replications of $Z_{2}$ from $Z_{2}|\theta_{2}=0.5∼N(0.5\times 125,125)$ where $V_{2}=125$ corresponds to approximately 500 patients with normally distributed responses. We then update each of the different prior distributions to find the set of 10,000 posterior distributions for each prior using a correlation of $\rho_{12}=0.8$ .

As before, we considered the definition of the PoS for a future study on A+B to be equal to the assurance for a two-sided superiority study with a planned sample size of 500 and a significance level of 0.05. We further assumed that, in order to run a study of combination A+B , we would need to observe a PoS of at least 0.6. The selection of an appropriate decision criterion on the PoS is discussed by Sabin et al.^25^ We recorded the PoS of each replication along with whether or not this would lead to a ‘go’ decision.

### 3.3 Results

The results of the simulation study are provided in Table 1. For the univariate approach, we do not need to consider multiple replications of a study on combination A+C , as we only consider direct information on combination A+B in this approach. Therefore, the mean PoS and the proportion of ‘go’ decisions actually correspond to the PoS and the ‘go’ decision based on the prior distribution as we observe no direct information on combination A+B in the simulation study, only indirect data on combination A+C , which is not considered in the univariate approach.

**Table 1.** Table showing the results for combination A+B of the simulation study where the true values of $\theta_{1}$ and $\theta_{2}$ are given by 0.5 and $\mu_{1}=0.2$ and $\mu_{2}$ represent the prior means for each combination.

	$\mu_{1}=0.2$	$\mu_{2}=0.2$	$\mu_{2}=0.5$	$\mu_{2}=0.8$
Univariate	Mean probability of success (PoS)	0.520	0.520	0.520
	% ‘Go’ ( PoS>0.6 )	0	0	0
Multivariate	Mean PoS	0.802	0.530	0.242
	% ‘Go’ ( PoS>0.6 )	99.6	22.7	0
Standard mixture	Mean PoS	0.661	0.525	0.381
	% ‘Go’ ( PoS>0.6 )	95.9	4.9	0
Hypothetical	Mean $\omega_{0}^{1}$	0.623	0.137	0.625
	Mean PoS	0.615	0.529	0.429
	% ‘Go’ ( PoS>0.6 )	74.9	18.6	0
Limiting	Mean $\omega_{0}^{1}$	0.639	0.153	0.642
	Mean PoS	0.610	0.529	0.434
	% ‘Go’ ( PoS>0.6 )	70.9	17.9	0

A correlation of 0.8 is used for the multivariate approaches. Note that the univariate approach does not update the distribution of combination A+B based on the results of combination A+C and that the ‘standard mixture’ approach refers to the mixture approach where the weights are not updated.

**Figure 4.**
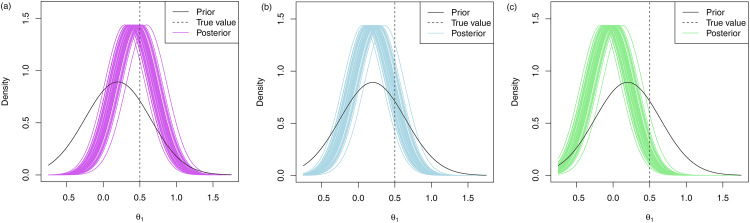
Marginal posterior distributions of $\theta_{1}$ for 50 of the 10000 replications using the multivariate approach. The prior mean for $\theta_{1}$ was set to $\mu_{1}=0.2$ and the prior mean for $\theta_{2}$ was set to (a) $\mu_{2}=0.2$ , (b) $\mu_{2}=0.5$ and (c) $\mu_{2}=0.8$ .

In Figure 4, 50 posterior distributions under the multivariate approach are plotted for fifty replications with the prior means given by $\mu_{1}=0.2$ and (a) $\mu_{2}=$ 0.2, (b) $\mu_{2}=$ 0.5 and (c) $\mu_{2}=$ 0.8.

We see that when we have a prior mean of $\mu_{1}=0.2$ , this leads to a PoS of 0.520 in the univariate approach, which does not exceed the required threshold to make a decision to run the next study. Therefore, under the ‘go’ rule of the PoS exceeding 0.6, if we do not use any indirect data, we will never run a study based on this univariate prior, despite the true value of $\theta_{1}$ being equal to 0.5.

**Figure 5.**
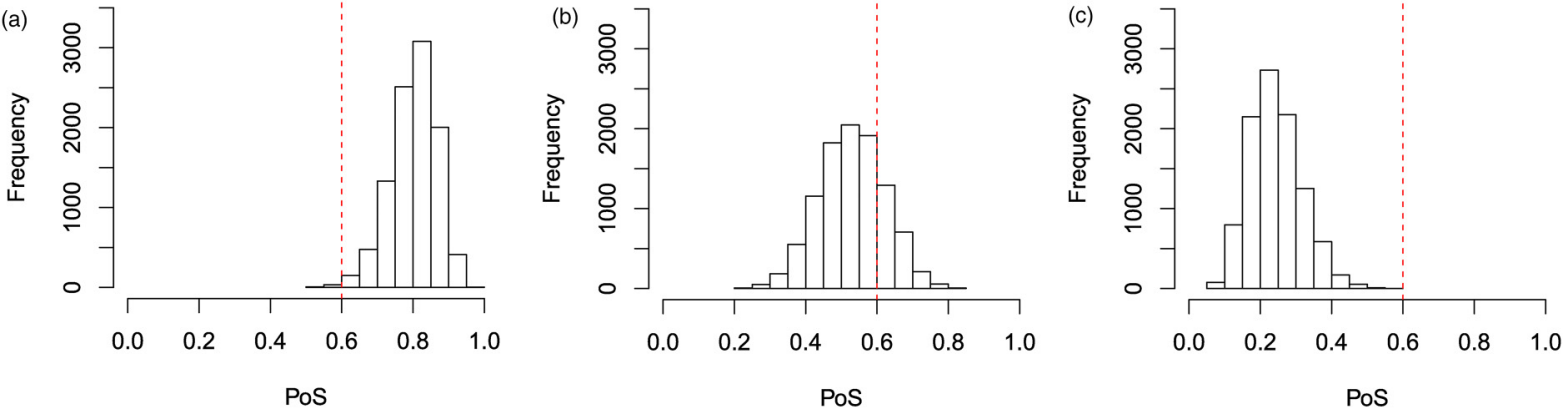
Histograms of the PoS for a study on $\theta_{1}$ in the simulation study using the multivariate approach. The prior mean for $\theta_{1}$ was set to $\mu_{1}=0.2$ and the prior mean for $\theta_{2}$ was set to (a) $\mu_{2}=0.2$ , (b) $\mu_{2}=0.5$ and (c) $\mu_{2}=0.8$ .

However, when we do include the indirect data, we make many more ‘go’ decisions. This, however, is also dependent on what the prior mean for combination A+C was. When $\mu_{2}=0.2$ , this is underestimating the true value of $\theta_{2}$ , therefore many of the observed studies will result in an estimate that exceeds the prior mean. This means that the posterior mean of combination A+C will be increased in the majority of cases and, since we have set $\rho_{12}=0.8$ , the posterior mean of combination A+B will also increase from a prior mean of $\mu_{1}=0.2$ . This will cause an overall increase in the PoS compared to when we did not include indirect data, hence we will choose to ‘go’ in the majority of cases. This is what we observe in Table 1, with the mean PoS being equal to 0.802 and the majority of the PoS values exceeding 0.6 resulting in 99.6% of decisions being ‘go’ decisions. This is also reflected in Figure 5(a), which provides a histogram of the success probabilities for this set of prior means.

When $\mu_{1}=0.2$ and $\mu_{2}=0.5$ , the majority of the replications will lead to little change in the posterior mean from the prior mean of combination A+C as the true treatment effect is given by $\theta_{2}=0.5$ . Therefore, this will cause the posterior mean of combination A+B to also remain similar to its prior mean as there is little difference between the data and our prior beliefs. However, since we will still be borrowing information, our posterior variance for combination A+B will decrease. This will cause the PoS to increase slightly compared to the univariate PoS. We observe a mean PoS of 0.530 and we would make the decision to run a study on combination A+B in 22.2% of cases, which is reasonably higher than had we not included the indirect information, despite the fact that our posterior mean for $\theta_{1}$ will still actually be an underestimate.

However, when our prior means for $\theta_{1}$ and $\theta_{2}$ underestimate and overestimate the truth, respectively, the multivariate method performs worse than the univariate method. This is because the data will cause the posterior mean for $\theta_{2}$ to reduce from $\mu_{2}=0.8$ to be closer to 0.5. This, in turn, will also cause the posterior mean of $\theta_{1}$ to decrease from its already low prior mean of $\mu_{1}=0.2$ . This leads to a mean PoS of 0.242 and zero ‘go’ decisions in all 10,000 replications. Consequently, if there is a chance that the prior estimates of the effects modelled may be incorrect in opposite directions, this methodology may not be appropriate. However, one might assume that there will be some correlation between the prior estimates of related combinations and so we might expect that they will often be incorrect in the same direction, given the nature of the problem. This is, in fact, what is assumed by the methodology presented here.

These results are also highlighted in the plots provided in Figure 4. In Figure 4(a), we see that when $\theta_{2}$ is underestimated by its prior mean, this leads to a posterior mean for $\theta_{2}$ that is on average higher than the prior mean. When $\theta_{2}$ is equal to its prior mean, on average the posterior mean is equal to the prior mean for $\theta_{1}$ as seen in Figure 4(b) and when the prior mean for $\theta_{2}$ is an overestimate, the posterior mean for $\theta_{1}$ is on average lower than the prior mean as seen in Figure 4(c).

These patterns will hold for other values of $\mu_{1}$ , $\mu_{2}$ and $\theta_{2}$ when the prior correlation is positive. When $\mu_{2}$ is an overestimate of the true value of $\theta_{2}$ , the mean of $\theta_{1}$ will decrease and cause a lower PoS than when $\mu_{2}$ is equal to the true value of $\theta_{2}$ . Similarly, when $\mu_{2}$ underestimates the true value of $\theta_{2}$ , the mean of $\theta_{1}$ will increase and cause a higher PoS than when $\mu_{2}$ is equal to the true value of $\theta_{2}$ . Results for our example with $\mu_{1}=0.5$ and $\mu_{1}=0.8$ can be found in Appendix B.

In this simulation study, we considered both $\theta_{1}$ and $\theta_{2}$ to be equal. If this was not the case, the patterns observed here would still be the same. That is, if we observe a value of $Z_{2}/V_{2}$ that is greater than the prior mean, $\mu_{2}$ , this will cause the posterior mean of $\theta_{1}$ to be greater than $\mu_{1}$ , assuming a positive prior correlation. Similarly, if we observe $Z_{2}/V_{2}<\mu_{2}$ , this would result in a posterior mean of $\theta_{1}$ that is less than $\mu_{1}$ . The size of the shift from the prior mean to the posterior mean of $\theta_{1}$ is related to the size of the difference between $Z_{2}/V_{2}$ and $\mu_{2}$ , the prior correlation, the observed study size and the prior variances.

**Figure 6.**
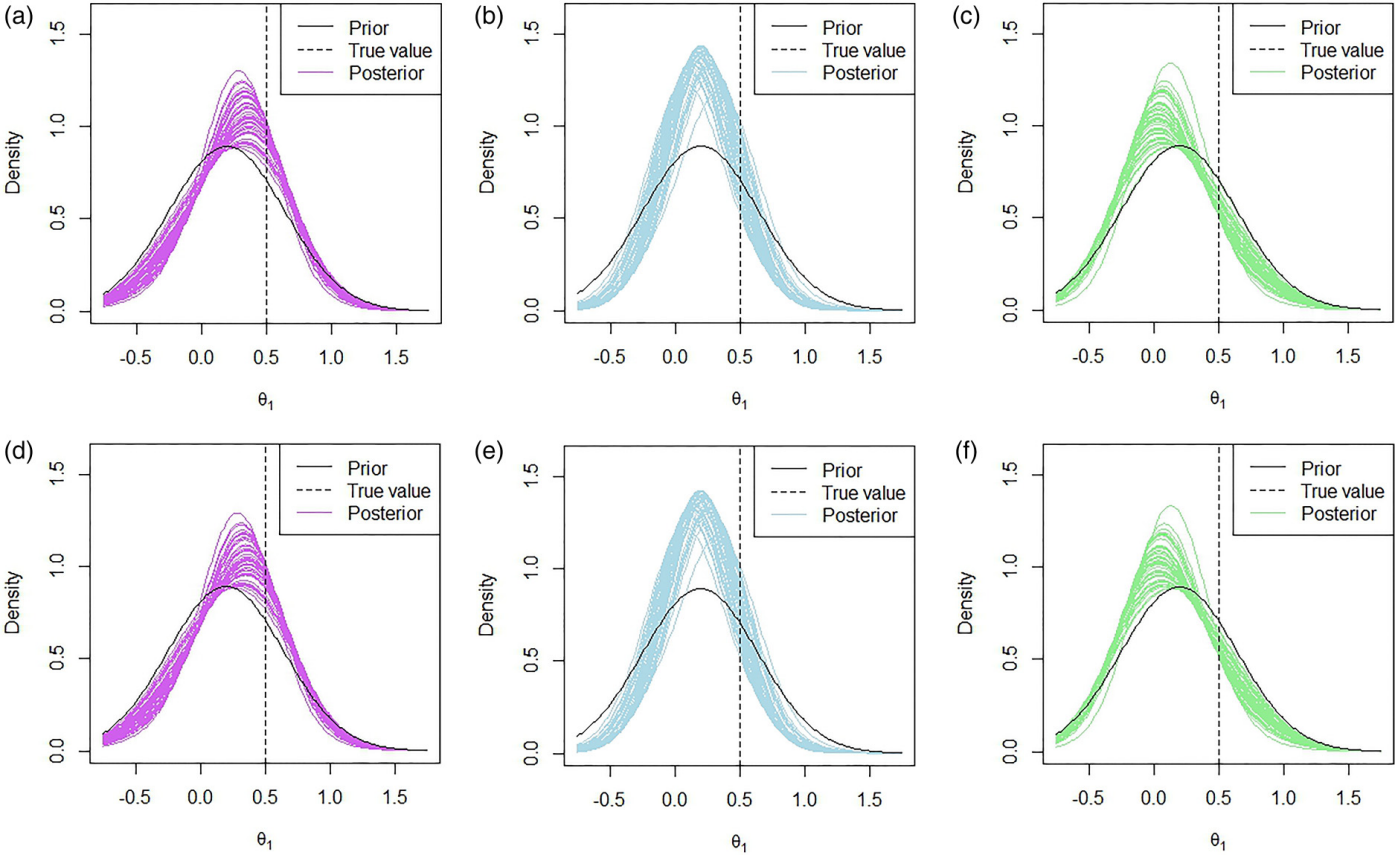
Marginal posterior distributions of $\theta_{1}$ for 50 of the 10000 replications using (a) to (c) the hypothetical posterior approach and (d) to (f) the limiting posterior approach. The prior mean for $\theta_{1}$ was set to $\mu_{1}=0.2$ and the prior mean for $\theta_{2}$ was set to (a,d) $\mu_{2}=0.2$ , (b,e) $\mu_{2}=0.5$ and (c,f) $\mu_{2}=0.8$ .

**Figure 7.**
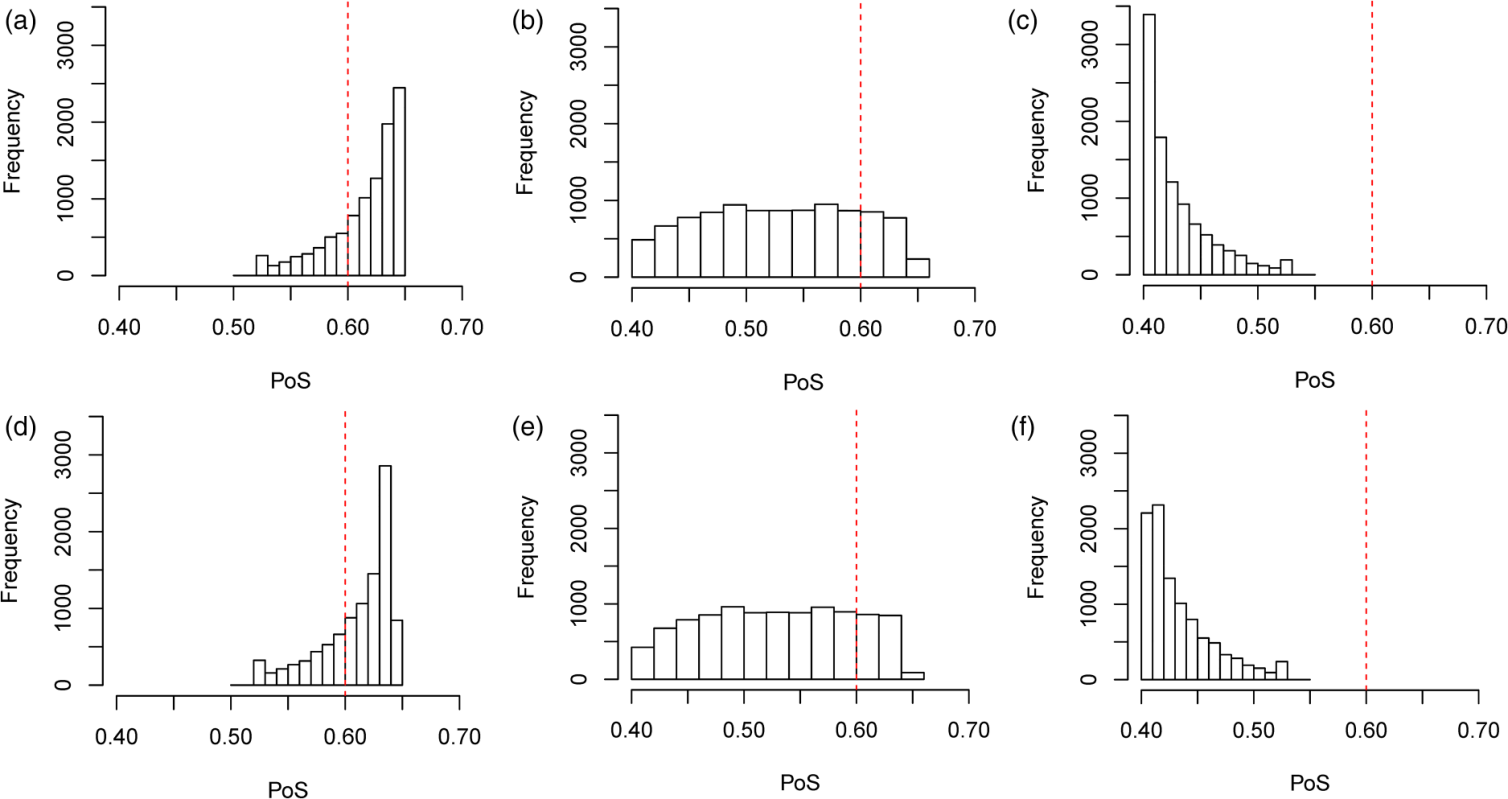
Histograms of the PoS for a study on $\theta_{1}$ in the simulation study using (a) to (c) the hypothetical posterior approach and (d) to (f) the limiting posterior approach. The prior mean for $\theta_{1}$ was set to $\mu_{1}=0.2$ and the prior mean for $\theta_{2}$ was set to (a,d) $\mu_{2}=0.2$ , (b,e) $\mu_{2}=0.5$ and (c,f) $\mu_{2}=0.8$ .

We also considered the performance of the hypothetical and limiting mixture posterior approaches that were presented in Section 2.4, along with the standard mixture approach in the simulation study and the results are presented in Table 1. The posterior distributions for the hypothetical and limiting posterior approaches are given in Figure 6 and the histograms of the success probabilities are given in Figure 7. We used prior weights of 0.5 for the correlated and uncorrelated components in each of the mixture approaches.

Since the mixture prior approach was introduced to account for the fact that two combinations may not be correlated and to borrow less when this is the case, the results that we see for the mixture approaches are not as extreme as in the multivariate approach in most cases. That is, the mean PoS and the proportion of ‘go’ decisions are lower in the mixture approach than in the multivariate approach when the indirect data causes an increase in mean, i.e. $\mu_{2}=0.2$ , and the mean PoS and the proportion of ‘go’ decisions are higher than in the multivariate approach when the indirect data causes a decrease in mean, i.e. $\mu_{2}=0.8$ . This is what we would hope to see given that when there is a large shift in means, a higher weight is assigned to an uncorrelated component of the model. In addition, the values of the mean PoS and the proportion of ‘go’ decisions for the mixture approaches in Table 1 lie between the values of the univariate and multivariate approaches. This is intuitive given that the mixture approaches are weighted mixtures of these two models hence the PoS of each replication will be bound by the univariate PoS and the multivariate PoS for that replication.

In Figure 6, we see very similar patterns in terms of the posterior distributions under the hypothetical and limiting posterior mixture approaches. One of the key observations from these posteriors is that the peak of the distributions is much closer to the prior means than in Figure 4, which showed the posteriors under the multivariate approach. This is due to the way in which we specified the weightings in Section 2.4.

One of the places where the effect of the mixture approaches is the most apparent is when $\mu_{1}=0.2$ and $\mu_{2}=0.8$ . Here, in most cases, evidence of a lower value of $\theta_{2}$ than was predicted by $\mu_{2}$ causes the posterior means of both $\theta_{1}$ and $\theta_{2}$ to decrease compared to their prior means resulting in a low mean PoS and no ‘go’ decisions in all 10,000 replications under the standard multivariate approach. In both mixture approaches, however, this shift from the prior to the posterior mean of $\theta_{2}$ caused the method to assign a higher posterior weight to the uncorrelated component of the mixture. This meant that the posterior mean did not drop as low as in the multivariate case, hence the mean PoS is much higher in the mixture approaches than in the multivariate approach. This is the situation where the mixture approaches may potentially provide a benefit over the standard multivariate approach.

The difference in the performance of the hypothetical posterior approach as compared to the limiting posterior approach is less obvious in Table 1. To learn more about the differences, we look at the histograms presented in Figure 7. In the histograms presented, we see that the limiting posterior approach is less likely to assign more extreme values of the PoS than the hypothetical posterior approach. In Figure 7(d), the final bar on the histogram is much smaller than those that precede it, despite an overall upwards trend until that point. This contrasts with what we see in Figure 7(a). Similarly, in Figure 7(c), we see that the first bar in the histogram is quite an amount higher than the first bar in the histogram in Figure 7(f). This is also reflected in Table 1 where we see that, in the simulation study, the limiting posterior approach assigns a higher posterior weight on average to the uncorrelated component of the model than the hypothetical posterior approach in all three cases.

The reason that the limiting posterior approach is less likely to assign these more extreme values is related to the way that the weights are assigned. In both the hypothetical and limiting posterior approaches, a value of p=1 means that the posterior under the multivariate approach is perfectly aligned with either the hypothetical or limiting posterior. In the simulation study, there is potential for values of p=1 in the hypothetical posterior approach. In fact, values of p=1 will always be possible no matter the size of the study. Even a study with only 20 patients could cause a weight of 1 to be assigned to the correlated component. However, the limiting posterior approach is more cautious in how it assigns the values of p . To have a value of p=1 , one would need a study that is large enough to result in a posterior variance equal to the limiting posterior variance. This means that the hypothetical posterior mixture approach has the potential to assign more extreme weights based on less information than the limiting posterior mixture approach would need to assign an equally extreme weighting. This is why we see the differences in the histograms for the success probabilities.

## 4 Discussion

In this paper, we have presented a method that allows the estimates of a set of related combination studies to be updated based on a single observation. This allows us to include both direct and indirect data in the treatment effect estimates, which allows us to reduce the variance and potentially improve the accuracy of these estimates. The probability distributions representing our beliefs about a particular therapy may often be used to gain an insight into the expected performance of a new therapy, but they are also often used to calculate the PoS of an upcoming study through the calculation of the assurance^4^ or other distribution based definitions of the probability of study success. One such framework would be the methodology presented by Ibrahim et al.,^16^ which uses a Bayesian approach to calculate the PoS for a trial based on current data and allows for the inclusion of covariates and patient characteristics in the calculation of the PoS. The PoS is often used to assist decision-making regarding the study. Improving the accuracy of treatment effect estimates may allow decision-making to improve by providing the decision-makers with the ability to recognise beneficial, or ineffective, treatments sooner.

The CLEOPATRA and MARIANNE studies were used to build an example that illustrates how the proposed methodology can be used in the real world. Identical marginal priors were used for both studies and the results of the CLEOPATRA study were used to update the joint distribution.

The methods presented provide an overall advantage over traditional univariate approaches due to the fact that they are able to use all available data. The scenario where these methods may not perform as well as the traditional methods are when the prior means of the treatment effects are incorrect by quite a substantial distance in opposite directions. However, we presented an extension to our method in Section 2.4 that allows this to be accounted for and limits the shift from the prior mean to the posterior mean when indirect data has caused the shift. It should be noted that, in some situations, such as if the observed treatment effect is much smaller than expected, it may be more conservative to update the treatment effect based on this indirect data than to decide not to consider the indirect data. Therefore, expert opinion should also be taken into account when considering the level of borrowing and whether the original multivariate approach or the robustification is most appropriate for the decision-makers.

In order to use the presented methods to calculate the PoS of an upcoming study based on the results of a related study, three types of information are required. The first is the significance level and the planned sample size of the upcoming study, both of which should be readily available if we are considering whether or not to run the study. The second is the score statistic and the Fisher information of the completed study, which should also be available at the conclusion of the study. The final type of information relates to the prior parameters for the distribution of treatment effects. The prior mean and variance for a treatment effect is a standard requirement when calculating the PoS. Rufibach et al.^26^ provided a discussion of the choice of prior when calculating the assurance and provides some recommendations. However, the prior correlation is an additional requirement of our approach over standard approaches. Rather than trying to quantify the correlation between treatments, one may instead consider this parameter as the amount of indirect information they would like to use (i.e. the strength of borrowing) when calculating the updated PoS as shown in Section 2.3. A potential area for further work is in the specification of the parameter and the potential to elicit this from the available data. We also presented a robustification that allows the alignment of our prior beliefs with the data to guide the degree of borrowing across combinations. The value of the PoS will always be dependent upon the prior parameters when we use methods such as the assurance^4^ to calculate it. Therefore, our method will naturally have some sensitivity towards the choices of these prior parameters and users should explore this when specifying prior parameters. However, since our method allows the user to incorporate relevant study data in this calculation, the PoS calculated under this approach will be less reliant upon the prior mean and variance than traditional univariate approaches.

We highlighted the performance of the multivariate method in Section 3 and showed that, compared to the univariate approach for calculating the PoS, it leads to improved decision-making regarding whether or not a particular combination study should be run.

A summary table of the approaches presented can be found in Table 2.

**Table 2.** Summary of the conclusions and recommendations of when to use each approach.

	Benefits	Limitations
Univariate	Well established/accepted	Cannot capture relationships
	No bias from indirect data	
Multivariate	Captures relationships	Relies on prior assumptions
	Considers indirect data	
Robustification	Captures relationships	Relies on prior assumptions
	Mixture of methods	

While most of the examples discussed and results presented were for a pair of combinations, it should be noted that the method can be used for any number of combinations. The method could also be used to assist internal decision-making based on external data. For example, multiple companies have developed PD-1/PD-L1 inhibitors, which are often combined with chemotherapies to treat different cancers. Companies could use the results of an external study to update the PoS of a study of their PD-1/PD-L1 inhibitor combination in the same indication. Furthermore, these methods could be applied not only in the setting of related combination studies but in different settings where there is potential to share information across studies. Some potential settings that could benefit from these methods include the same combination but in different indications and programmes in different regions.

## Supplemental Material

sj-R-1-smm-10.1177_09622802231151218 - Supplemental material for Updating the probability of study success for combination therapies using related combination study data Click here for additional data file. Supplemental material, sj-R-1-smm-10.1177_09622802231151218 for Updating the probability of study success for combination therapies using related combination study data by Emily Graham, Chris Harbron and Thomas Jaki in Statistical Methods in Medical Research

## Appendix A: methodology

### A.1 Kalman filter

The Kalman Filter is used to consider a dynamic system that we are able to take measurements from and we will consider this system at discrete time intervals. There is often noise within measurements and inputs but we can use filtering to gain information about the state of the system given previous measurements. The method that we will describe here is described in further detail by Anderson and Moore.^22^

Let $x_{k}$ be the state of the system at time k and let $y_{k}$ be a measurement of the system taken at time k that includes noise. We can write

$$x_{k+1}=F_{k}x_{k}+G_{k}w_{k}$$

where $w_{k}∼MVN(0,Q_{k})$ describes the input noise and **F** is the state transition matrix. We can write the measurement at time point k as

$$y_{k}=H_{k}^{T}x_{k}+v_{k}$$

where $v_{k}∼MVN(0,R_{k})$ describes the output noise.

We are interested in estimating $x_{k}$ given the observations $y_{0},\ldots ,y_{k}$ . In order to simplify the problem and the equations required to perform the filtering, Anderson and Moore^22^ modifies the problem to first consider the prediction of $x_{k}$ given $y_{0},\ldots ,y_{k-1}$ before considering the prediction of $x_{k}$ given $y_{0},\ldots ,y_{k}$ .

We will use the subscript k|j to denote time point k based upon everything up until time point j . Then the equations used to make estimates of the state of the system at time k under the Kalman filter are given in Anderson and Moore^22^ as follows.

$$\begin{array}{rl} {\hat{\mu }}_{0|-1} & =\mu_{0}\\ {\hat{\Sigma }}_{0|-1} & =\Sigma_{0}\\ {\hat{\mu }}_{k+1|k} & =\left(F_{k}-K_{k}H_{k}^{T}\right){\hat{\mu }}_{k|k-1}+K_{k}y_{k}\\ K_{k} & =F_{k}{\hat{\Sigma }}_{k|k-1}H_{k}{\left(H_{k}^{T}{\hat{\Sigma }}_{k|k-1}H_{k}+R_{k}\right)}^{-1}\\ {\hat{\Sigma }}_{k+1|k} & =F_{k}\left[{\hat{\Sigma }}_{k|k-1}-{\hat{\Sigma }}_{k|k-1}H_{k}{\left(H_{k}^{T}{\hat{\Sigma }}_{k|k-1}H_{k}+R_{k}\right)}^{-1}H_{k}^{T}{\hat{\Sigma }}_{k|k-1}\right]F_{k}^{T}+G_{k}Q_{k}G_{k}^{T}\\ {\hat{\mu }}_{k|k} & ={\hat{\mu }}_{k|k-1}+{\hat{\Sigma }}_{k|k-1}H_{k}{\left(H_{k}^{T}{\hat{\Sigma }}_{k|k-1}H_{k}+R_{k}\right)}^{-1}\left(y_{k}-H_{k}^{T}{\hat{\mu }}_{k|k-1}\right)\\ {\hat{\Sigma }}_{k|k} & ={\hat{\Sigma }}_{k|k-1}-{\hat{\Sigma }}_{k|k-1}H_{k}{\left(H_{k}^{T}{\hat{\Sigma }}_{k|k-1}H_{k}+R_{k}\right)}^{-1}H_{k}^{T}{\hat{\Sigma }}_{k|k-1} \end{array}$$

Let us now apply this to our problem. In our problem, the ‘state’ of the system is the vector of true treatment effects θ . This vector does not evolve in time and it does not have any noise, therefore for our system $F_{k}=I$ and $G_{k}=0$ .

Suppose that we observe an outcome on combination A+C , as before, which we will summarise using the score statistic $Z_{2}$ and Fisher information $V_{2}$ . Then, in the Kalman filter setting our observation will be $Z_{2}$ and we can write this in terms of θ as

$$Z_{2}=\left(0,V_{2}\right)\theta +\epsilon$$

where ϵ is our output noise and has distribution $\epsilon ∼N(0,V_{2})$ . Hence, for our system $H_{k}=(0,V_{2}{)}^{T}$ , $v_{k}=\epsilon$ and $R_{k}=V_{2}$ .

Applying the above formulae for the Kalman Filter to our problem, we get the following.

$$\begin{array}{rl} {\hat{\mu }}_{0|-1} & =\mu_{0}\\ {\hat{\Sigma }}_{0|-1} & =\Sigma_{0}\\ {\hat{\mu }}_{0|0} & ={\hat{\mu }}_{0|-1}+{\hat{\Sigma }}_{0|-1}{\left(0,V_{2}\right)}^{T}{\left(\left(0,V_{2}\right){\hat{\Sigma }}_{0|-1}{\left(0,V_{2}\right)}^{T}+V_{2}\right)}^{-1}\left(Z_{2}-\left(0,V_{2}\right){\hat{\mu }}_{0|-1}\right)\\ & =\mu_{0}+\Sigma_{0}{\left(0,V_{2}\right)}^{T}{\left(\left(0,V_{2}\right)\Sigma_{0}{\left(0,V_{2}\right)}^{T}+V_{2}\right)}^{-1}\left(Z_{2}-\left(0,V_{2}\right)\mu_{0}\right)\\ & =\left(\begin{array}{c} \mu_{1}-\frac{\rho_{12}\sigma_{1}\sigma_{2}V_{2}}{1+V_{2}\sigma_{2}^{2}}\mu_{2}+\frac{\rho_{12}\sigma_{1}\sigma_{2}}{1+V_{2}\sigma_{2}^{2}}Z_{2}\\ \frac{1}{1+V_{2}\sigma_{2}^{2}}\mu_{2}+\frac{\sigma_{2}^{2}}{1+V_{2}\sigma_{2}^{2}}Z_{2} \end{array}\right)\\ {\hat{\Sigma }}_{0|0} & ={\hat{\Sigma }}_{0|-1}-{\hat{\Sigma }}_{0|-1}{\left(0,V_{2}\right)}^{T}{\left(\left(0,V_{2}\right){\hat{\Sigma }}_{0|-1}{\left(0,V_{2}\right)}^{T}+V_{2}\right)}^{-1}\left(0,V_{2}\right){\hat{\Sigma }}_{0|-1}\\ & =\Sigma_{0}-\Sigma_{0}{\left(0,V_{2}\right)}^{T}{\left(\left(0,V_{2}\right)\Sigma_{0}{\left(0,V_{2}\right)}^{T}+V_{2}\right)}^{-1}\left(0,V_{2}\right)\Sigma_{0}\\ & =\left(\begin{array}{cc} \sigma_{1}^{2}-\frac{V_{2}\rho_{12}^{2}\sigma_{1}^{2}\sigma_{2}^{2}}{1+V_{2}\sigma_{2}^{2}} & \frac{\rho_{12}\sigma_{1}\sigma_{2}}{1+V_{2}\sigma_{2}^{2}}\\ \frac{\rho_{12}\sigma_{1}\sigma_{2}}{1+V_{2}\sigma_{2}^{2}} & \frac{\sigma_{2}^{2}}{1+V_{2}\sigma_{2}^{2}} \end{array}\right) \end{array}$$

This is the same result that we saw in Section 2.3.

### A.2 Gaussian Markov Random Fields

We will now formulate the problem using Gaussian Markov Random Fields.^23^ A Gaussian Markov Random Field (GMRF) is a finite dimensional random vector $x=(x_{1},\ldots ,x_{n+1}{)}^{T}$ that follows the multivariate normal distribution and has some additional Markovian properties to be defined. First, let us write

$$x∼\mathrm{MVN}(\mu ,Q^{-1})$$

where μ is the mean vector and Q is the precision matrix.

Recall that two variables, $x_{i}$ and $x_{j}$ , are conditionally independent given another variable, $x_{k}$ , if and only if

$$\pi (x_{i},x_{j}|x_{k})=\pi (x_{i}|x_{k})\pi (x_{j}|x_{k})$$

where π(⋅) represents a probability density function. We can represent the conditional independence structure of x using a graph G=(N,E) . In this graph, the nodes, N , represent the random variables, $x_{1},\ldots ,x_{n}$ , and the edges, E , provide us with information regarding the conditional independence structure. We can say that x is a GMRF with respect to G if and only if

$$\pi (x)=(2\pi {)}^{-(n+1)/2}|Q{|}^{1/2}\exp\;\left(-\frac{1}{2}(x-\mu {)}^{T}Q(x-\mu )\right)$$

and

$$Q_{ij}\neq 0\;\Leftrightarrow \;(i,j)\in E\;\;\forall \;\;i\neq j$$

The properties of GMRFs that are of particular use for our problem are the conditional properties. First, let us partition the random vector into two sets $x=(x_{A},x_{B}{)}^{T}$ . We can similarly partition the mean vector, $\mu =(\mu_{A},\mu_{B}{)}^{T}$ , and the precision matrix,

$$Q=\left(\begin{array}{cc} Q_{AA} & Q_{AB}\\ Q_{BA} & Q_{BB} \end{array}\right).$$

Then, it can be shown that the conditional distribution of $x_{A}|x_{B}$ is also a GMRF with respect to the subgraph $G^{A}=(N^{A},E^{A})$ . $N^{A}$ is a subset of N containing the nodes that represent the variables in $x_{A}$ . $E^{A}$ is a subset of E containing the edges between the nodes in $N^{A}$ . The mean and precision matrix of this conditional distribution are given, respectively, by

(5)
$$\mu_{A|B}=\mu_{A}-Q_{AA}^{-1}Q_{AB}\left(x_{B}-\mu_{B}\right)\;\mathrm{and}\;Q_{A|B}=Q_{AA}.$$

Hence, we can write

(6)
$$x_{A}|x_{B}∼\mathrm{MVN}\left(\mu_{A|B},Q_{A|B}^{-1}\right).$$

Let us now formulate our problem in the setting of GMRFs. We will illustrate the method using our two drug example, but will also provide the results for n combinations. In our setting, the random variables are the treatment effects of the different combinations alongside the score statistic for the study that is going to be run. We can write this as $(\theta ,Z_{i}{)}^{T}$ where we will be observing the outcome of a study on combination i .

We will also need to consider the conditional independence structure of this vector of random variables. For our simple example of combinations A+B and A+C , $\theta_{1}$ and $Z_{2}$ will be conditionally independent given $\theta_{2}$ . This represents the beliefs that $\theta_{1}$ and $\theta_{2}$ are ‘related’ and the score statistic, $Z_{2}$ , from a study on combination 2 is dependent only on the value of $\theta_{2}$ .

Suppose that we are going to observe the outcome of a study on A+C , which we will summarise using $Z_{2}$ and $V_{2}$ . Under the assumption that $Z_{2}$ is approximately normally distributed, $Z_{2}∼N(V_{2}\theta_{2},V_{2})$ , we can write

$$Z_{2}=V_{2}\theta_{2}+\epsilon_{2}\;\mathrm{where}\;\epsilon_{2}∼N(0,V_{2}).$$

Using this formulation, we are able to find the expectation and variance of $Z_{2}$ in this framework and the covariance between $Z_{2}$ and θ . We can then write our GMRF as

$$\left(\begin{array}{c} \theta_{1}\\ \theta_{2}\\ Z_{2} \end{array}\right)∼\mathrm{MVN}\left(\left(\begin{array}{c} \mu_{1}\\ \mu_{2}\\ V_{2}\mu_{2} \end{array}\right),\left(\begin{array}{ccc} \sigma_{1}^{2} & \rho_{12}\sigma_{1}\sigma_{2} & V_{2}\rho_{12}\sigma_{1}\sigma_{2}\\ \rho_{12}\sigma_{1}\sigma_{2} & \sigma_{2}^{2} & V_{2}\sigma_{2}^{2}\\ V_{2}\rho_{12}\sigma_{1}\sigma_{2} & V_{2}\sigma_{2}^{2} & V_{2}^{2}\sigma_{2}^{2}+V_{2} \end{array}\right)\right).$$

The precision matrix, Q , of this GMRF is given by

$$\begin{array}{rl} Q & =\left(\begin{array}{cc} Q_{\theta \theta } & Q_{\theta Z_{2}}\\ Q_{Z_{2}\theta } & Q_{Z_{2}Z_{2}} \end{array}\right)\\ & =\left(\begin{array}{ccc} \frac{1}{\sigma_{1}^{2}(1-\rho_{12}^{2})} & \frac{-\rho_{12}}{\sigma_{1}\sigma_{2}(1-\rho_{12}^{2})} & 0\\ \frac{-\rho_{12}}{\sigma_{1}\sigma_{2}(1-\rho_{12}^{2})} & \frac{1}{\sigma_{2}^{2}(1-\rho_{12}^{2})}+V_{2} & -1\\ 0 & -1 & \frac{1}{V_{2}} \end{array}\right). \end{array}$$

Note that if we have n combinations and we observe a study on combination i then we can find the mean vector and covariance matrix in the same way and the GMRF for this problem would be given by

$$\left(\begin{array}{c} \theta_{1}\\ \vdots \\ \theta_{n}\\ Z_{i} \end{array}\right)∼\mathrm{MVN}\left(\left(\begin{array}{c} \mu_{1}\\ \vdots \\ \mu_{n}\\ V_{i}\mu_{i} \end{array}\right),\left(\begin{array}{cccc} \sigma_{1}^{2} & \cdots & \rho_{1n}\sigma_{1}\sigma_{n} & V_{i}\rho_{1i}\sigma_{1}\sigma_{i}\\ \vdots & \ddots & \vdots & \vdots \\ \rho_{1n}\sigma_{1}\sigma_{n} & \cdots & \sigma_{n}^{2} & V_{i}\rho_{in}\sigma_{i}\sigma_{n}\\ V_{i}\rho_{1i}\sigma_{1}\sigma_{i} & \cdots & V_{i}\rho_{in}\sigma_{i}\sigma_{n} & V_{i}^{2}\sigma_{i}^{2}+V_{i} \end{array}\right)\right).$$

Using the conditional properties of GMRFs, we are able to find the conditional distribution of $\theta |Z_{i}$ . Applying equations (5) and (6) as given in Rue and Held,^23^ we find that

$$\theta |Z_{i}=z_{i}∼\mathrm{MVN}\left(\mu_{\mathrm{post}},\Sigma_{\mathrm{post}}\right)$$

where

$$\mu_{\mathrm{post}}=\mu -Q_{\theta \theta }^{-1}Q_{\theta Z_{i}}(z_{i}-V_{i}\mu_{i})\;\mathrm{and}\;\Sigma_{\mathrm{post}}=Q_{\theta \theta }^{-1}.$$

For our simple example containing combinations A+B and A+C , this gives

$$\left(\begin{array}{c} \theta_{1}\\ \theta_{2} \end{array}\right)|Z_{2}∼\mathrm{MVN}\left(\left(\begin{array}{c} \mu_{1}-\frac{\rho_{12}\sigma_{1}\sigma_{2}V_{2}}{1+V_{2}\sigma_{2}^{2}}\mu_{2}+\frac{\rho_{12}\sigma_{1}\sigma_{2}}{1+V_{2}\sigma_{2}^{2}}z_{2}\\ \frac{1}{1+V_{2}\sigma_{2}^{2}}\mu_{2}+\frac{\sigma_{2}^{2}}{1+V_{2}\sigma_{2}^{2}}z_{2} \end{array}\right),\left(\begin{array}{cc} \sigma_{1}^{2}-\frac{V_{2}\rho_{12}^{2}\sigma_{1}^{2}\sigma_{2}^{2}}{1+V_{2}\sigma_{2}^{2}} & \frac{\rho_{12}\sigma_{1}\sigma_{2}}{1+V_{2}\sigma_{2}^{2}}\\ \frac{\rho_{12}\sigma_{1}\sigma_{2}}{1+V_{2}\sigma_{2}^{2}} & \frac{\sigma_{2}^{2}}{1+V_{2}\sigma_{2}^{2}} \end{array}\right)\right).$$

This is the same result that we saw in Section 2.3.

**Table 3.** Table showing the results for combination A+B of the simulation study where the true values of $\theta_{1}$ and $\theta_{2}$ are given by 0.5 and $\mu_{1}=0.5$ and $\mu_{2}$ represent the prior means for each combination.

	$\mu_{1}=0.5$	$\mu_{2}=0.2$	$\mu_{2}=0.5$	$\mu_{2}=0.8$
Univariate	Mean probability of success (PoS)	0.762	0.762	0.762
	% ‘Go’ ( PoS>0.6 )	100	100	100
Multivariate	Mean PoS	0.968	0.861	0.621
	% ‘Go’ ( PoS>0.6 )	100	100	60.2
Hypothetical	Mean $\omega_{0}^{1}$	0.623	0.137	0.625
	Mean PoS	0.837	0.848	0.725
	% ‘Go’ ( PoS>0.6 )	100	100	100
Limiting	Mean $\omega_{0}^{1}$	0.639	0.153	0.642
	Mean PoS	0.833	0.846	0.727
	% ‘Go’ ( PoS>0.6 )	100	100	100

Note that the univariate approach does not update the distribution of combination A+B based on the results of combination A+C .

### Appendix B: Results

**Table 4.** Table showing the results for combination A+B of the simulation study where the true values of $\theta_{1}$ and $\theta_{2}$ are given by 0.5 and $\mu_{1}=0.8$ and $\mu_{2}$ represent the prior means for each combination.

	$\mu_{1}=0.8$	$\mu_{2}=0.2$	$\mu_{2}=0.5$	$\mu_{2}=0.8$
Univariate	Mean probability of success (PoS)	0.915	0.915	0.915
	% ‘Go’ ( PoS>0.6 )	100	100	100
Multivariate	Mean PoS	0.998	0.982	0.906
	% ‘Go’ ( PoS>0.6 )	100	100	100
Hypothetical	Mean $\omega_{0}^{1}$	0.623	0.137	0.625
	Mean PoS	0.946	0.973	0.919
	% ‘Go’ ( PoS>0.6 )	100	100	100
Limiting	Mean $\omega_{0}^{1}$	0.639	0.153	0.642
	Mean PoS	0.945	0.972	0.919
	% ‘Go’ ( PoS>0.6 )	100	100	100

Note that the univariate approach does not update the distribution of combination A+B based on the results of combination A+C .
